# ERp19 contributes to tumorigenicity in human gastric cancer by promoting cell growth, migration and invasion

**DOI:** 10.18632/oncotarget.3649

**Published:** 2015-03-26

**Authors:** Jing Wu, Xue-hua Chen, Xin-qiong Wang, Yi Yu, Jian-min Ren, Yuan Xiao, Tong Zhou, Pu Li, Chun-di Xu

**Affiliations:** ^1^ Department of Pediatrics, Ruijin Hospital, Shanghai Jiao Tong University School of Medicine, Shanghai, People's Republic of China; ^2^ Shanghai Key Laboratory of Gastric Neoplasms, Shanghai Institute of Digestive Surgery, Ruijin Hospital, Shanghai Jiao Tong University School of Medicine, Shanghai, People's Republic of China

**Keywords:** ERp19, gastric cancer, tumorigenicity, FAK, paxillin

## Abstract

ERp19, a mammalian thioredoxin-like protein, plays a key role in defense against endoplasmic reticulum stress. It belongs to the protein disulfide isomerize (PDI) family, whose members have been implicated in development of breast, ovarian and gastrointestinal cancers. However, the role of ERp19 in gastric cancer (GC) remains undefined. Therefore, we sought to investigate the expression and prognostic value of ERp19 in GC patients, and to explore the role of ERp19 in tumorigenicity. Expression of ERp19 in gastric tissues was assessed by immunohistochemical staining and real-time PCR in clinical samples of GC patients. Statistical analysis of clinical cases revealed that the expression levels of ERp19 were higher in tumor tissues than non-tumor tissues. And the level of ERp19 expression was correlated with tumor size, lymph node involvement and poor clinical prognosis. Furthermore, ERp19 knockdown dramatically suppressed gastric cancer cell growth, inhibited cellular migration/invasion and down-regulated the phosphorylation of FAK and paxillin, whereas ERp19 over-expression reversed these changes. We conclude that ERp19 contributes to tumorigenicity and metastasis of GC by activating the FAK signaling pathway, and may function as an oncogene in GC. ERp19 may represent a new diagnostic and prognostic marker and a novel target for the treatment of GC.

## INTRODUCTION

Gastric cancer (GC) is the fourth most common malignancy and the second leading cause of cancer related death worldwide. Half of all GC cases occur in Eastern Asia [1-3], where most patients are diagnosed at an advanced stage of GC [4]. Despite the increasing efficacy of surgical treatments and adjuvant therapy, nearly 60% of those patients affected succumb to GC [5]. Additionally, GC is a heterogeneous disease, and prognosis is difficult to predict from histological analysis. Tumor progression is thought to be controlled by multiple factors at multiple stages involving the activation of oncogenes or inactivation of tumor suppressor genes. However, promising molecules being useful for GC early diagnosis and targeted therapy are still limited. Therefore, elucidating the molecular mechanisms responsible for GC carcinogenesis has the potential to highlight valuable prognostic markers and targets for treatment of this disease.

Protein disulfide isomerase (PDI) family proteins are emerging as important players in carcinogenesis. PDIA1 has been found to be highly expressed in lymphoma [6-8], kidney [9-11], prostate [12, 13] and lung [14] tumors. In addition, PDIA1 [15, 16], AGR2 [17], TXNDC5 [18] were reported to support tumor survival and progression. Abnormal regulation of PDI family proteins was also found in several gastric malignancies. Tsuji *et al*. showed that AGR2 secreted from gastric signet-ring cell carcinoma (SRCC) cells plays important roles in the progression of gastric SRCC by affecting the surrounding fibroblasts [19]. Zhang *et al.* suggested that TXNDC5 could promote the growth, proliferation and invasion of gastric cancer cells [18]. Leys *et al.* found that ERp57 expression is down-regulated in gastric adenocarcinoma and correlated with depth of invasion, TNM stage of tumors and patient survival [20]. Although the relationship between PDI family and cancer has been gradually understood in recent years, the functions and underlying mechanisms of PDI family members were still limited and have yet to be clearly defined.

A member of PDI family proteins, ERp19, which contains a NH(2)-terminal signal peptide and a thioredoxin (Trx) domain is known by several names including: Txndc12, AGR1, ERp16, ERp18, hAG-1, PDIA16 and hTLP19 [21]. ERp19 is ubiquitously expressed in all tissues, and especially abundant in the liver and placenta [22]. In Hela cells, ERp19 expression inhibits induction of apoptosis by agents including brefeldin A, tunicamycin, and dithiothreitol, while depletion of ERp19 by RNA interference enhanced apoptosis in response to these agents [23]. DU145, a prostate cancer cell line was also found to express ERp19. In comparison to CD44- DU145 cells, ERp19 was up-regulated in CD44+ DU145 cells that possess stemness and tumorigenicity [24]. Additionally, using whole-genome expression microarrays, expression of ERp19 was detected in non-tumor lung tissue from lung adenocarcinoma patients, and potentially associated with the patients' survival [25]. These clues suggest that ERp19 contributes to tumorigenesis, however the precise role of ERp19 in GC remain unclear.

In this study we examined the expression level of ERp19 in gastric carcinoma tissues and corresponding non-tumor mucosa tissues. Furthermore, we evaluated the association between ERp19 expression and clinical features, as well as the duration of patient survival. We found that ERp19 is likely an oncogene in GC. ERp19 promotes GC cell growth, migration and invasion, and may contribute to the tumorigenicity of GC via the FAK/paxillin and ERK1/2 pathways.

## RESULTS

### ERp19 is overexpressed in gastric cancer tissues and GC cells

**Figure 1:**
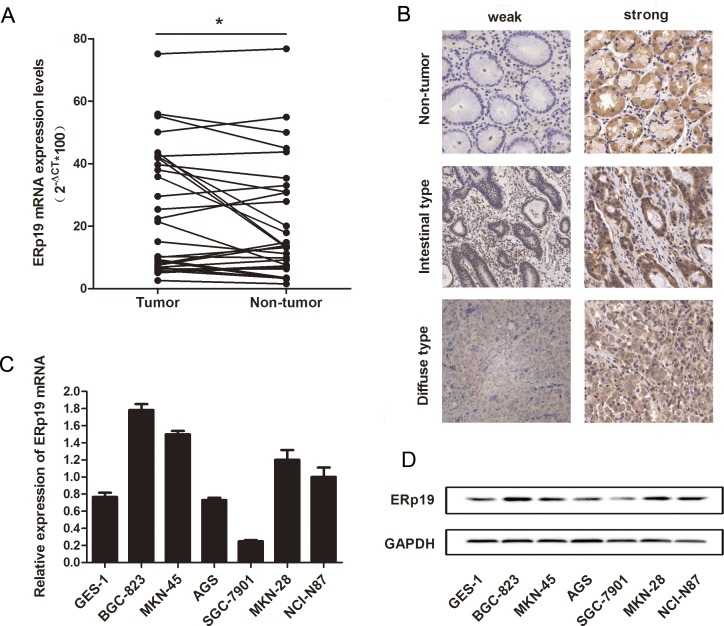
Expression of ERp19 in gastric cancer tissues and cell lines **A**, Expression of ERp19 mRNA in 29 gastric cancer tissues was analyzed by qRT-PCR. Data is shown as 2^−ΔCt^ (*P < 0.05). **B**, Expression of ERp19 was performed with Immunohistochemical (IHC) staining in non-tumor gastric tissues, intestinal-type gastric cancer, and diffuse-type gastric cancer tissues. Original magnification: ×200. **C** and **D**, Expression of ERp19 in human gastric cancer cell lines. The mRNA and protein levels of ERp19 were detected by qRT-PCR and western blotting respectively.

ERp19 expression was initially evaluated in human gastric cancer and matched adjacent non-tumor tissues. We assessed the level of ERp19 expression in 29 patients with gastric cancer by qRT-PCR, and found that level of ERp19 mRNA in gastric cancer tissues was significantly higher than in non-tumor tissues (*P*=0.0352) (Fig. 1A). In addition, expression of ERp19 protein in GC tissue microarray sections, obtained from 90 individuals, 67 men and 23 women, age range 41-83 years was assessed by immunohistochemical staining. We found that ERp19 was expressed in the cytoplasm of gastric carcinoma cells (Fig. 1B). Of the 180 specimens, ERp19 staining was detected in 57.78 % (52 of 90) of gastric cancers, but significantly fewer in adjacent non-tumor tissues (37.78%, 34 of 90) (*P*=0.007). To validate these findings in GC cancer cell lines, we used qRT-PCR and western blot analysis to examine ERp19 expression in gastric cancer cell lines and normal gastric mucosal epithelial cell line (GES-1). In comparison to GES-1, the expression of ERp19 RNA and protein was higher in BGC-823, MKN-45, MKN-28, NCI-N87, but lower in AGS, SGC-7901 (Fig. 1C-D). These findings indicate that ERp19 was overexpressed in gastric cancer tissues and most GC cell lines However, whether ERp19 expression was correlated with clinicopathological features remained to be determined.

### ERp19 expression level is correlated with clinicopathological features and survival rate in GC patients

**Figure 2:**
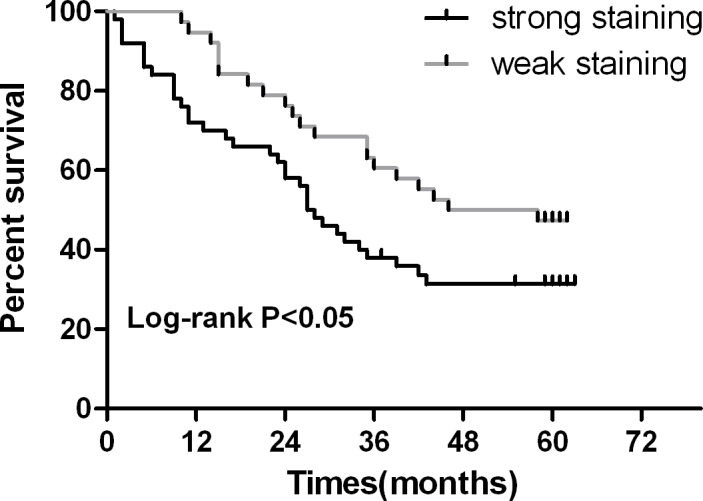
Kaplan-Meier survival curves in gastric carcinoma according to ERp19 staining Patients with ERp19 strong staining had a significantly poorer prognosis than those with weak staining. *P* < 0.05.

Association between ERp19 protein expression and clinicopathological characteristics of GC in 90 patients was shown in Table 1. Chi-square test suggested that high expression of ERp19 in GC was significantly correlated with tumor size (*P*=0.041) and lymph node involvement (*P*=0.034). However, there were no statistically significant relationships between ERp19 expression and other clinicopathological variables such as age, gender, histological grade, Lauren's classification or TNM stage. 5-year-follow-up of the 90 patients revealed that 61% (55 of 90) patients had died, with the median survival time of 27.5 months for patients with strong ERp19 staining and 52 months for those with weak staining. The Kaplan-Meier survival analysis indicated that the survival rate of patients with strong staining was significantly lower than that of those with weak staining (*P* < 0.05, Fig. 2). Together these results provide evidence that up-regulated ERp19 expression may be associated with GC malignancy.

**Table 1:** Relationship between ERp19 expression level and clinicopathological variables in 90 GC patients

Clinicopathological variable	No. of patients	ERp19 staining		*P* value
		Weak	Strong	
Normal tissue	90	56	34	0.007*
Gastric carcinoma	90	38	52	
Age (years)				0.423
<60	29	14	15	
≽60	61	24	37	
Gender				0.728
Male	67	29	38	
Female	23	9	14	
Tumor size (cm)				0.041*
≼5	34	19	15	
>5	56	19	37	
Histological grade				0.584
Well, Moderately	24	9	15	
Poorly, undifferentiated	66	29	37	
Lauren's classification				0.108
Intestinal-type	56	20	36	
Diffuse-type	34	18	16	
Lymph node involvement				0.034*
Absence	25	15	10	
Presence	65	23	42	
TNM stage				0.433
I+II	36	17	19	
III+IV	54	21	33	

Immunohistochemistry (IHC) scores were calculated by multiplying the percentage of positive cells (0 is <5% positive cells, 1 is 5%-25% positive cells, 2 is 25%-50% positive cells, 3 is 50%-75% positive cells and 4 is >75% positive cells) by stain intensity (0 is no staining, 1 is weak staining, 2 is moderate staining and 3 is strong staining) in five different high power fields for each section. The IHC score of 0-3 was defined as weak expression and 4-12 as strong expression.

### ERp19 promotes cell growth *in vitro* and *in vivo*

Given that ERp19 is significantly up-regulated in GC, it may act as an oncogene. In order to investigate the function of ERp19 in GC cell lines, we chose the ERp19 high-expression cell line BGC-823 and low-expression cell line SGC-7901 for further studies. shRNA targeting ERp19 was transfected into BGC-823 cells to generate a model of ERp19-knockdown (BGC-823/ERp19 shRNA), while an ERp19-expressing lentivirus vector was transfected into SGC-7901 cells to generate a model of ERp19 overexpression (SGC-7901/ERp19).

**Figure 3:**
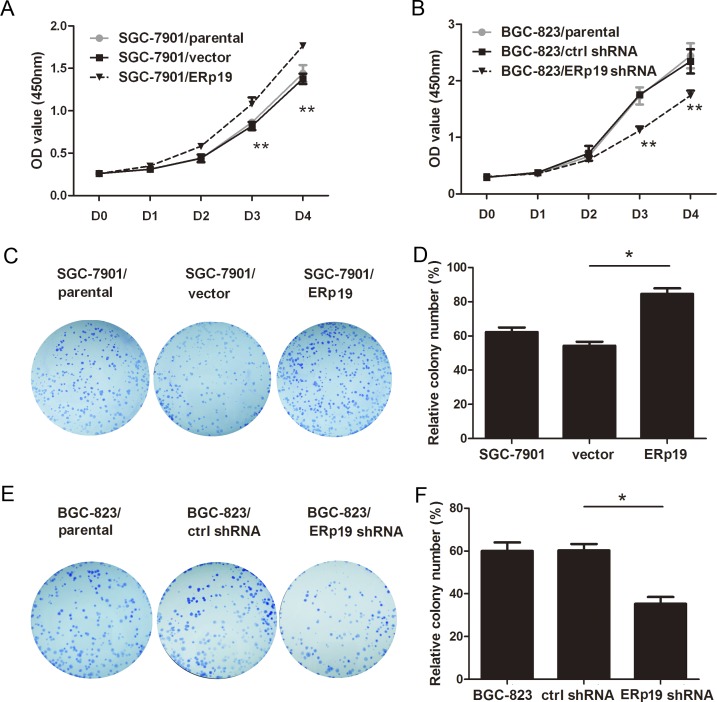
Effects of ERp19 on cell growth in human gastric cancer cells **A** and **B**, Effects of ERp19 overexpression and knockdown on cell growth using CCK8 assay. (*P < 0.05, **P < 0.01). **C** and **D**, Effects of ERp19 overexpression on SGC-7901 cell growth using the plate colony formation assay. The same amounts of SGC-7901/parental, SGC-7901/vector and SGC-7901/ERp19 cells were plated into a 6-well plate. Cell colonies were stained and counted on the 14th day (*P < 0.05). **E** and **F**, Effects of ERp19 knockdown on BGC-823 growth using the plate colony formation assay. The data represents mean ± SD of three independent experiments (*P < 0.05).

**Figure 4:**
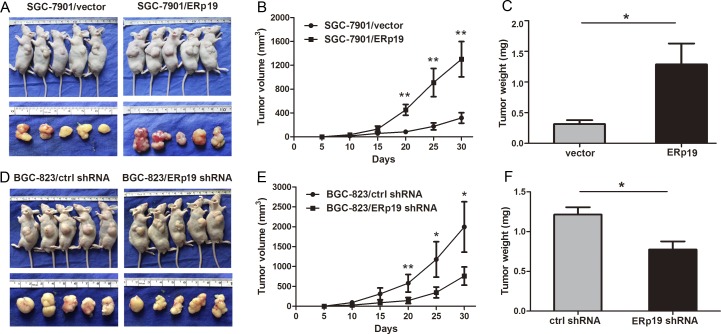
Effects of ERp19 on tumor growth in vivo **A** and **D**, Representative photographs of tumors derived from SGC7901/vector, SGC7901/ERp19, BGC-823/ctrl shRNA and BGC-823/ERp19 shRNA cells, respectively. **B** and **E**, The growth curves of tumors after injection of GC7901/vector, SGC7901/ERp19, BGC-823/ctrl shRNA and BGC-823/ERp19 shRNA cells in nude mice (*P < 0.05, **P < 0.01; n=5 per group). Tumor sizes were measured every 5 days during the 30 days. **C** and **F**, Average weights of tumors derived from SGC7901/vector, SGC7901/ERp19, BGC-823/ctrl shRNA and BGC-823/ERp19 shRNA cells in nude mice (*P < 0.05). Data are shown as the mean ± SD.

To examine the role of ERp19 in GC cell growth, we first evaluated cell proliferation by CCK8 assay. As shown in Fig. 3A and 3B, overexpression of ERp19 promoted proliferation, and ERp19 knockdown significantly inhibited cell proliferation. In addition, up-regulation of ERp19 had no effect on proliferation of GES-1 (Fig. S1). Colony formation assays revealed that SGC-7901/ERp19 formed more colonies than control and parental cells (*P* < 0.05, Fig. 3C and 3D; *P* < 0.01, Fig. S2A and S2B). Consistently, ERp19 knockdown dramatically suppressed colony formation of BGC-823 cells, in comparison to parental cells and controls (*P*<0.05, Fig. 3E and 3F; *P*<0.05 Fig. S2C and S2D). These findings indicate that ERp19 promotes human GC cell growth and proliferation *in vitro*. We finally wanted to know whether ERp19 could further affect the tumorigenicity *in vivo*. SGC7901/vector, SGC7901/ERp19, BGC-823/ctrl shRNA and BGC-823/ERp19 shRNA were subcutaneously injected into the nude mice and tumor formation was monitored. On day 30, mice were sacrificed under anesthesia and tumor weights were measured. Tumors grew faster in SGC7901/ERp19 and BGC-823/ctrl shRNA groups compared to the groups of SGC7901/vector and BGC-823/ERp19 shRNA, respectively (Fig. 4A, 4B, 4D and 4E). Furthermore, tumor weights were higher in SGC7901/ERp19 group than that in the group of SGC7901/vector (1.29±0.76 g vs. 0.31± 0.15 g, *P* < 0.05, Fig. 4C). As expected, compared to BGC-823/ctrl shRNA group, the weight of tumors derived from BGC-823/ERp19 shRNA group was much lower (1.21±0.21 g vs. 0.77±0.23 g, *P* < 0.05, Fig. 4F). These data suggest that ERp19 could enhance the cell growth *in vivo.*

### ERp19 enhances the migration and invasion of GC cells

**Figure 5:**
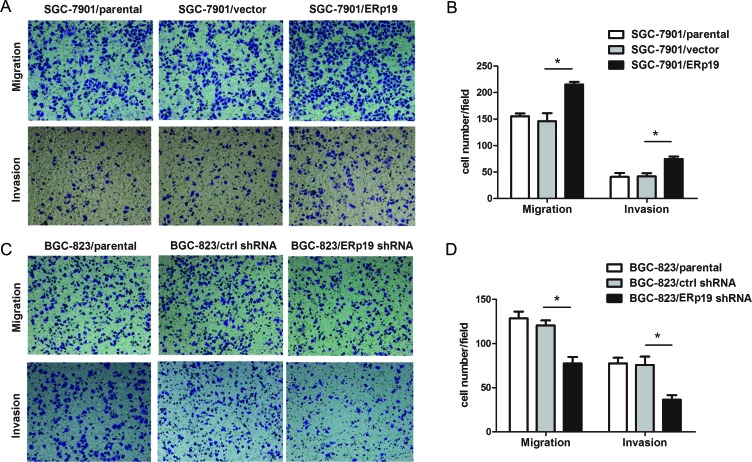
Effects of ERp19 on cell migration and invasion in human gastric cancer cells **A**, Representative images of migrated/invaded SGC-7901 cells through chambers' membrane. **B**, The mean number of migrated/invaded cells in SGC7901/parental, SGC7901/vector and SGC7901/ERp19 groups. Cell numbers were counted in five randomly selected microscopic fields (*P < 0.05). **C**, Representative images of migrated/invaded BGC-823cells through chambers' membrane. **D**, The mean number of migrated/invaded cells in BGC-823/parental, BGC-823/ctrl shRNA and BGC-823/ERp19 shRNA groups. Cell numbers were counted in five randomly selected microscopic fields (*P < 0.05). The data is shown as mean ± SD of three independent experiments.

To further characterize the effect of ERp19, we investigated the migration and invasion of transfected cells *in vitro* by transwell assays. The number of cells migrating through the chamber in SGC7901/ERp19 (215.25±10.31) was significantly higher than cells transfected with SGC7901/parental (155.25±11.12) and SGC7901/vector (146±30.34) (Fig. 5A and 5B). The same result was also observed in parallel invasion assays with SGC7901/ERp19 (74.75±9.22), SGC7901/parental (41±14.45), and SGC7901/vector (41.75±12.28) transfected cells (Fig. 5A and 5B). Inversely, ERp19 shRNA transfected BGC-823 cells apparently suppressed cell migration and invasion ability. (Migration assay: BGC-823/ERp19 shRNA group: 77.5±14.53, BGC-823/parental group: 128.5±15.29, BGC-823/ctrl shRNA group: 120.5±11.39; Invasion assay: BGC-823/ERp19 shRNA group: 36.5±10.02, BGC-823/parental group: 77.5±12.87, BGC-823/ctrl shRNA group: (75.75±18.86) (Fig. 5C and 5D).

### ERp19 promotes the phosphorylation of FAK/paxillin and ERK1/2

**Figure 6:**
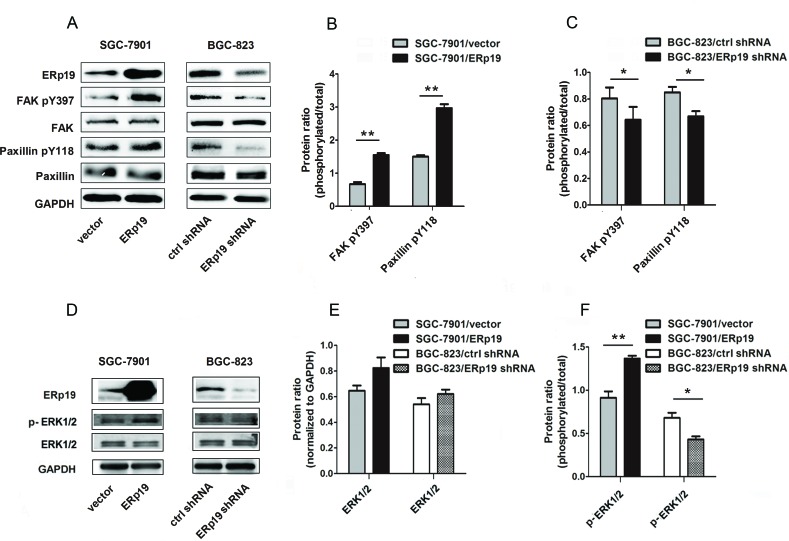
Effects of ERp19 on the phosphorylation levels of FAK/paxillin and ERK1/2 in gastric cancer cells **A**, Effects of ERp19 on FAK and paxillin phosphorylation levels were analyzed by western blotting. GAPDH was used as a loading control. **B**, Protein ratio of FAK Tyr397 and paxillin Tyr118 in SGC-7901 cells (**P < 0.01). **C**, Protein ratio of FAK Tyr397 and paxillin Tyr 118 in BGC-823 cells (*P < 0.05). **D**, Effects of ERp19 on ERK1/2 phosphorylation levels were analyzed by western blotting. GAPDH was used as a loading control. **E**, Protein ratio of ERK1/2 in GC cells. **F**, Protein ratio of p-ERK1/2 in GC cells (*P < 0.05; **P < 0.01).

**Figure 7:**
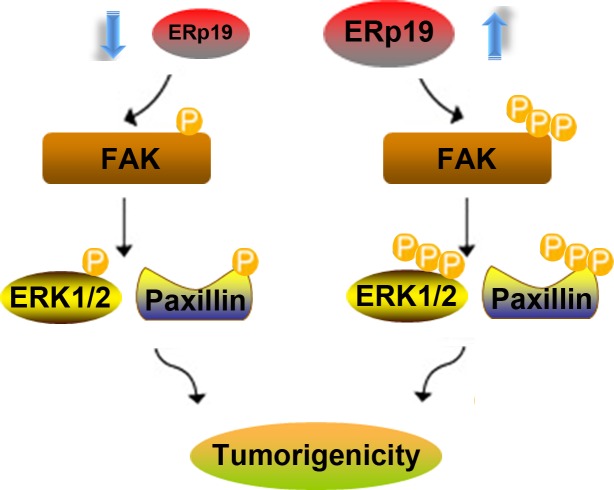
A hypothetical schematic of the contribution of ERp19 to GC cells via activation of the FAK/paxillin and ERK1/2 pathways

Accumulating evidence reveals that tumor invasion and metastasis may be regulated by FAK/paxillin pathway [27]. We thus investigated whether ERp19 could affect FAK/paxillin phosphorylation in SGC-7901 and BGC-823 cells. The efficacy of ERp19 knockdown and overexpression is shown in Fig. S3 and Fig. 6A. Over 70% of ERp19 protein was suppressed in BGC-823/ERp19 shRNA cells in comparison to BGC-823/ctrl shRNA cells, while the protein level was significantly higher in SGC-7901/ERp19 cells than SGC-7901/vector cells (Fig. 6A; Fig. S3A and S3B). As expected, ERp19 overexpression in SGC-7901 cells markedly enhanced FAK phosphorylation at Tyr-397 and paxillin phosphorylation at Tyr118 (Fig. 6A and 6B). In contrast, phosphorylation of these proteins was inhibited by knockdown of ERp19 in BGC-823 cells (Fig. 6A and 6C). Extracellular Signal-Regulated Kinase (ERK) activation and signaling have been reported to be involved in cancer cell growth and proliferation [28]. We thus sought to assess whether ERp19 mediates its effects via ERK1/2 signaling. Assessment of ERK1/2 phosphorylation revealed that the phosphorylation of ERK1/2 was up-regulated in SGC-7901/ERp19 cells while down-regulated in BGC-823/ERp19 shRNA cells (Fig. 6D-F). These findings suggest that ERp19 may be as an upstream molecule to stimulate activation of FAK/paxillin and ERK1/2, contributing to tumorigenicity of human gastric cancer cells (Fig. 7).

## DISCUSSION

ERp19 is a member of the PDI gene family and contains seven exons spanning more than 35 kb [22]. Recently, PDI and its homologs have been reported to have an impact on carcinogenesis [16, 29]. However, the aberrant expression of ERp19 in gastric cancer, and its potential role in GC cells remain largely unknown. In the present study, ERp19 expression is substantially up-regulated in GC tissues compared to adjacent non-tumor tissues according to immunohistochemical staining and real-time PCR, indicating that ERp19 may contribute to tumorigenicity in GC. In addition, we statistically analyze the pathological features and postoperative survival of clinical cases with gastric carcinoma, and found that tumor with high ERp19 expression had inclinations towards larger tumor size and more extensive lymph node metastasis. Patients with high ERp19 expression had significantly shorter postoperative survival periods. These data indicated ERp19 may be associated with GC proliferation and metastasis. Thus, all the above suggest the great clinical and potential research significance of ERp19 in gastric carcinoma.

According to our findings, ERp19 may function as an oncogene, causing malignant progression in GC cells. In CCK8 and clonogenic assays, overexpression of ERp19 promotes proliferation and colony formation of GC cells. *In vivo* results from nude mice models supported the experimental outcomes *in vitro*. Consistently, ERp19 knockdown inhibits the growth of GC cells. These findings indicated that ERp19 could enhance gastric tumorigenesis by regulating cell growth. In transwell assays, we discovered that ERp19 overexpression was correlated with increased GC cell migration and invasion, and ERp19 knockdown was correlated with decreased GC cell migration and invasion, suggesting that ERp19 may further promote gastric tumorigenesis by enhancing GC cell motility.

In addition to ERp19, another member of PDI family, ERp29, has been previously studied in breast cancer and showed correlations to tumor cell growth and survival. The study further indicated that overexpression of ERp29 attenuates the expression of FAK and p-FAK [30]. In our study, we demonstrated that ERp19 expression significantly correlated with phosphorylation levels of FAK. FAK is a cytoplasmic protein tyrosine kinase (PTK) that is localized to cellular focal adhesions. It is tyrosine phosphorylated in response to a variety of stimuli including integrin and growth factor receptors. The PI3K-Akt and MEK-ERK1/2 pathways, activated by FAK, have been reported to control the altered growth of tumor cells [31-32]. Constitutive activation of the ERK1/2 pathway has been implicated in a variety of human tumors, including renal cell, metastatic esophagogastric, breast carcinomas and leukemia, highlighting the potential involvement of cellular ERK1/2 proteins in tumorigenesis [33-34]. We found that ERp19 significantly increased the growth and proliferation of GC cells in parallel with up-regulating FAK phosphorylation at Tyr397 and ERK1/2 phosphorylation at Thr202/Tyr204, indicating that ERp19 could control cell proliferation by regulating the ERK1/2 pathway. In addition to enhancing proliferation, FAK, in combination with paxillin, was also thought to influence migration/invasion of tumors through modulation of peripheral actin structures and cell-cell adhesions [35]. Recent studies show that elevated FAK expression, phosphorylation and catalytic activity were frequently associated with increased rates of both migration and invasion [36-37].

The phosphorylation of FAK at Tyr397 was found in invasive ovarian carcinomas, but not in normal epithelium [38]. However, the role and underlying mechanisms of FAK in GC progression remains to be unclear. In this study we investigated the role of ERp19 in regulating the levels of FAK protein and phosphorylation. We showed, for the first time, that FAK phosphorylation was higher in a GC cell model of ERp19-overexpression, while lower in a GC cell model of ERp19-knockdown, indicating that ERp19 could activate FAK leading to enhanced migration and invasion of human GC cells. Phosphorylation/dephosphorylation events are the key steps in the signal transduction pathway. Our results suggest that ERp19 overexpression or knockdown could increase or decrease FAK phosphorylation at Tyr397 and paxillin phosphorylation at Tyr118, respectively. FAK activation leads to the autophosphorylation of FAK at Tyr397, creating a high affinity binding site for the SH2 domain of Src family kinases. Src binding to FAK promotes increased Src kinase activity, and subsequently leads to phosphorylation and activation of FAK and its downstream signals [39]. Paxillin is a substrate for the FAK-Src complex and paxillin phosphorylation generates binding sites for the SH2/SH3 adaptor protein Crk, which influences the activity of downstream signal molecules including Rac1, PAK, and so contributes to cell motility [40]. Previous studies have indicated the important roles of Crk, Rac1 and PAK in tumorigenesis. RNA interference targeting the Crk gene has been shown to inhibit migration and invasion of human cancer cells [41, 42]. Deregulated expression and activity of Rac1were observed in a variety of tumor cells and may be associated with a number of malignancy-related processes, including proliferation, angiogenesis, invasion and metastasis [43]. Similarly, PAKs are increased in many human cancers and play key roles in oncogenic signaling [44]. As our study indicated a role of ERp19 in promoting the migration and invasion of GC cells, we speculate that ERp19 may do so via activating the FAK/paxillin and ERK1/2 pathways.

In conclusion, our studies indicate that ERp19 expression is up-regulated in gastric cancer tissues, and is associated with poor clinical outcomes. In addition, ERp19 promotes GC cell proliferation, migration and invasion. The latter function may be achieved partly by regulating FAK/paxillin and ERK1/2 pathways. Taken together, our findings indicate that ERp19 may serve as a novel target for clinical diagnosis and treatment of GC.

## MATERIALS AND METHODS

### Patient samples

29 GC patients who underwent radical resection were recruited randomly from Ruijin Hospital between 2006 and 2008. The tissue samples from those patients were confirmed by pathological diagnosis. The corresponding non-tumor location was at least 6 cm from the gastric tumor. All the specimens including tumor and paired non-tumor tissues were placed in liquid nitrogen after resection and stored at −80°C until RNA extraction. The study was approved by the Shanghai Jiao Tong University Medical School institutional review board and written informed consent was obtained from all participants.

### Cell lines

The six gastric cancer cell lines BGC-823, MKN-45, AGS, SGC-7901, MKN-28 NCI-N87 and immortalized normal gastric epithelial cell line GES-1 were preserved by Shanghai Digestive Surgery Institute. Cell lines were cultured in RPMI 1640 medium supplemented with 10% FBS (Gibco).

### Plasmids construction and transfection

ERp19 shRNA (sc-60597-SH) or control shRNA (sc-108060) plasmids (Santa Cruz Biotechnology) were transfected into gastric cancer cells using Lipofectamine 2000 (Invitrogen). ERp19 cDNA ORF was cloned into the pHBLV-IRES-ZsGreen-PGK-Puro plasmid (HanbioTM) for lentivirus production. Stable cell lines were screened by purimycine and identified by western blotting.

### Tissue microarray and immunohistological analysis

Gastric cancer tissue arrays were purchased from the National Engineering Center for BioChips in Shanghai, China. After being dewaxed, hydrated and blocked of non-specific binding sites, the microarray was incubated with 1:150 rat monoclonal anti-ERp19 antibody (Abcam) at 4°C overnight and 1:100 secondary biotinylated anti-rabbit antibody for 10 min at 37°C. Finally, sections were developed with DAB solution and counterstained with haematoxylin.

### Quantitative realtime-PCR (qRT-PCR)

Total RNA was extracted using Trizol Reagent kit (Invitrogen), and cDNA was synthesized using the Reverse Transcription kit (Takara). ERp19 primers were as follows: sense 5′- TGGCAAGGTGCATCCTGAAAT-3′ and antisense 5′-TGCTCGGCACTGACATAAAAA-3′; GAPDH were used as internal control reference: sense 5′-TTGGCATCGTTGAGGGTCT-3′, antisense 5′-CAGTG GGAACACGGAAAGC-3′. PCR amplification was performed using SYBR Green PCR master mix kit. A melting-curve analysis was performed to check the specificity of the amplified PCR products.

### Western blot analysis

Cell lysates were prepared with RIPA. Protein in the supernatant was extracted, and its concentration was measured using the BCA Protein Assay Kit. An equal amount (50 ug) of total cellular protein was electrophoresed by denaturing 12.5% SDS-PAGE and transferred to 0.22um polyvinylidene difluoride (PVDF) membranes (Millipore, MA, USA). The locations of proteins of interest were detected by primary antibodies for overnight at 4°C. ERp19 antibody was from Abcam;GAPDH antibody was from Kangchen Bio-tech; FAK, FAK pY397, paxillin, paxillin pY118, ERK1/2 and p-ERK1/2 antibodies were from Cell Signaling Biotechnology. After HRP conjugated-secondary antibody bound to the primary antibodies, the proteins were visualized using enhanced chemiluminescence (ECL) reagent.

### Cell proliferation assay

Cells were seeded onto 96-well plates at a ﬁnal density of 2.0×10^3^ viable cells/well and incubated for 4 days. Cell proliferation was then measured by colorimetric water-soluble tetrazolium salt (WST) assay using a cell counting kit CCK-8. OD450 was measured 2 h after adding CCK-8 at 0, 24, 48, 72 and 96 h.

### Colony formation assay

In plate colony formation assay, cells were resuspended in RPMI 1640 containing 10% FBS and layered onto 6-well plates at 5×10^2^ cells/well. The cells were incubated for 2 weeks and stained with crystal violet. Colonies containing 50 cells or more were counted.

In soft agar colony formation assay, cells were resuspended with 0.3% soft agar in RPMI 1640 containing 20% FBS and layered onto 0.6% solidified agar in RPMI 1640 containing 10% FBS in 6-well plates at 1×10^3^ cells/well. The cells were incubated for 2 weeks and stained with MTT. Colonies containing 50 cells or more were counted.

### Cell migration and invasion assay

Cell migration and invasion was measured using transwell chamber (8 μm, 24-well format; Corning, Lowell, MA, USA). To measure migration, 2×10^5^ cells were resuspended in 0.2 ml of serum-free medium and added to the upper chamber, and 0.6 ml of medium containing 10% FBS was added to the lower chamber. Cells were incubated for 24 hours. To measure invasion, diluted Matrigel (BD Biosciences) was used to coat the insert chambers' membrane. Cells were cultured for 48 h under the same conditions. Finally, cells that migrated or invaded into the lower chambers were fixed with methanol, stained with crystal violet and counted in six random fields.

### In vivo tumorigenesis

SPF grade male BALB/c nude mice were purchased from Institute of Zoology, Chinese Academy of Sciences. 2×10^6^ cells were resuspended in 0.2ml of RPMI 1640 and subcutaneously injected into 4-week-old male nude mice. The length (L) and width (W) of each tumor were measured every 5 days with calipers, and the volume was calculated using the formula: (W+L)/2×W×L×0.5236 [26].

### Statistical analysis

Statistical analyses were performed using SPSS 13.0 software. The relationship between the ERp19 expression level and clinicopathologic parameters were calculated with the Pearson χ2 test. Survival curves were explored by Kaplan–Meier method, and differences between two groups were evaluated by the log-rank test. Comparisons were performed by Student *t* test (two groups) or one-way ANOVA (multiple groups). *P* < 0.05 was considered statistically significant.

## SUPPLEMENTARY MATERIAL, FIGURES


