# Supplementary material for: ERp19 contributes to tumorigenicity in human gastric cancer by promoting cell growth, migration and invasion
Source: Oncotarget. 2015 Mar 26;6(14):11794–805. doi: 10.18632/oncotarget.3649 (PMC4494905; doi:10.18632/oncotarget.3649)
Supplement: Supplementary file 1 [file oncotarget-06-11794-s001.pdf]

ERp19 contributes to tumorigenicity in human gastric cancer by promoting cell growth, migration and invasion

Supplementary Material

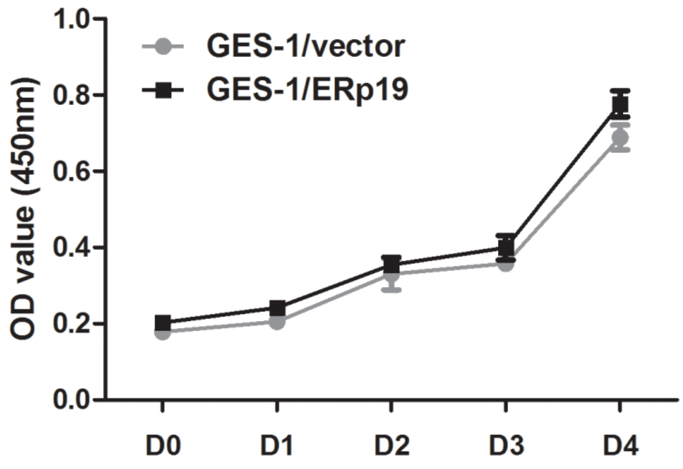

Fig.S1: Effects of ERp19 on the ability of cell growth in GES-1.

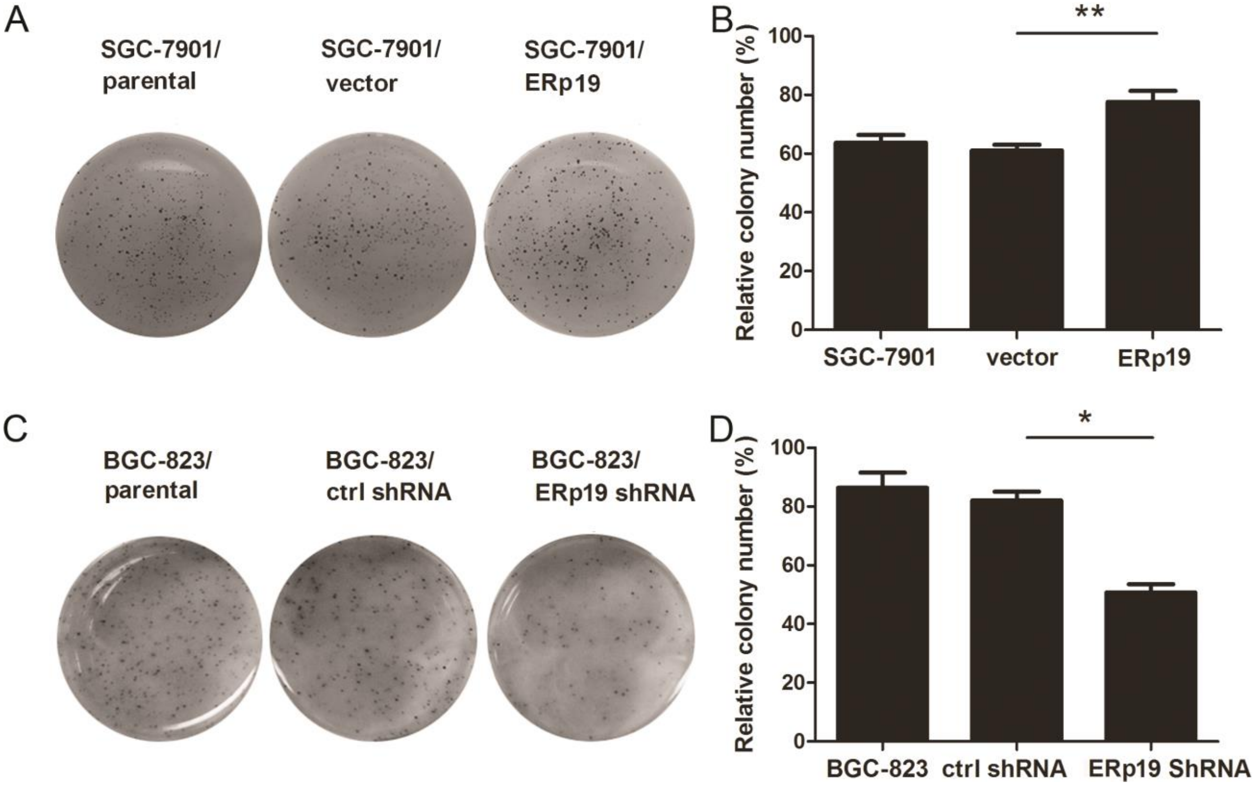

Fig.S2: Effects of ERp19 on cell growth in human gastric cancer cells using the soft agar colony formation assay.

A and B Effects of ERp19 overexpression on SGC-7901 cell growth using the soft agar colony formation assay. The same amounts of SGC-7901/parental, SGC-7901/vector and SGC-7901/ERp19 cells were plated into a 6-well plate. Cell colonies were stained and counted on the 14th day (**$P<0.01$). C and D, Effects of ERp19 knockdown on BGC-823 growth using the soft agar colony formation assay. The data represents mean ± SD of three independent experiments (*$P<0.05$).

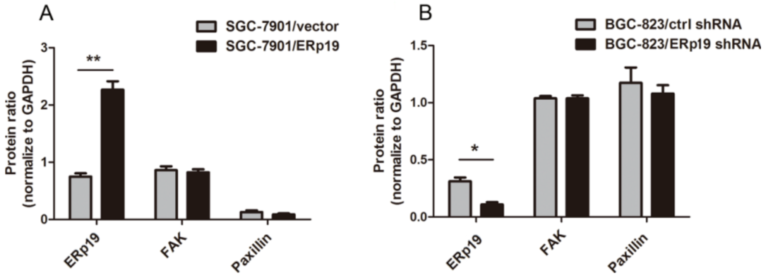

Fig.S3: Protein ratio of ERp19, FAK and paxillin normalized to GAPDH. A, Protein ratio of ERp19, FAK and paxillin in SGC-7901 cells (***P*<0.01). B, Protein ratio of ERp19, FAK and paxillin in BGC-823 cells (**P*<0.05).
